# Liver-specific deletion of *Agpat5* protects against liquid sucrose-induced hyperinsulinemia and glucose intolerance

**DOI:** 10.1016/j.bbrc.2024.151059

**Published:** 2024-11-23

**Authors:** Sylwia Michorowska, Kara R. Vogel, Raghav Jain, Samantha L. St Clair, Judith A. Simcox, Brian W. Parks

**Affiliations:** a Department of Nutritional Sciences, University of Wisconsin-Madison, Madison, WI, USA; b Department of Biochemistry, University of Wisconsin-Madison, Madison, WI, USA; c Department of Drug Chemistry, Pharmaceutical, and Biomedical Analysis, Faculty of Pharmacy, Medical University of Warsaw, Poland; d Howard Hughes Medical Institute, Department of Biochemistry, University of Wisconsin-Madison, Madison, WI, USA

**Keywords:** Agpat5, Glycerophospholipid, Insulin, Sucrose, Diabetes, MASLD

## Abstract

Agpat5 (1-acylglycerol-3-phosphate O-acyltransferase 5) is a broadly expressed lipid regulatory enzyme involved in glycerophospholipid metabolism. Multiple genetic studies in mice and humans have identified that *Agpat5* is associated with plasma insulin, cholesterol, and alanine aminotransferase levels. Despite the strong genetic evidence on *Agpat5*, no study has investigated its liver-specific role in physiology. Here, we conducted a series of metabolic studies under four distinct dietary conditions to assess the impact of liver-specific *Agpat5* deletion on plasma insulin levels, glucose tolerance, plasma cholesterol levels, and hepatic steatosis. Liver-specific deletion of *Agpat5* did not affect plasma insulin levels, glucose tolerance, plasma cholesterol levels, or hepatic steatosis in mice fed a chow diet, high-fat diet, or Western diet. However, when mice consumed a chow diet combined with liquid sucrose, liver-specific deletion of *Agpat5* resulted in significantly decreased plasma insulin levels and improved glucose tolerance without alterations in body weight or fat mass. Using global lipidomics, we identified that *Agpat5* specifically modulated levels of phosphatidylglycerol and cardiolipin within the livers of mice consuming liquid sucrose. Overall, our findings indicate a liver-specific role of *Agpat5* in contributing to hyperinsulinemia and glucose tolerance in the absence of body weight changes when consuming liquid sucrose.

## 1. Introduction

The acylglycerophosphate acyltransferase (AGPAT) family of enzymes regulates glycerophospholipid metabolism by acylating lysophosphatidic acid (LPA) at the *sn*-2 position, generating phosphatidic acid (PA) [1]. They are involved in the *de novo* glycerophospholipid and triacylglycerol biosynthetic pathways, contributing to cellular membrane composition and as precursors to lipid mediators. AGPATs exhibit distinct but overlapping tissue-specific gene expression patterns and show acyl specificity related to chain length and unsaturation [2]. Of the 11 members of the AGPAT family of enzymes, *Agpat1–5* show specificity to produce PA from LPA [3]. Among the AGPAT enzymes, *Agpat5* (1-Acylglycerol-3-Phosphate O-Acyltransferase 5) is unique in its cellular localization, substrate specificity, and genetic data supporting its role in metabolic disease. Localized to the outer mitochondrial membrane, *Agpat5* acylates substrates beyond LPA, such as lysophosphatidylethanolamine (LPE), lysophosphatidylcholine (LPC), lysophosphatidylinositol (LPI), and lysophosphatidylserine (LPS) [4]. In mice, *Agpat5* was identified within a genome-wide significant quantitative trait locus (QTL) associated with plasma insulin levels [5]. Another study in mice identified *Agpat5* within a genome-wide significant QTL associated with hypoglycemia-induced glucagon secretion [6]. In human genome-wide association studies (GWAS), *AGPAT5* is located within a genome-wide significant locus associated with plasma levels of alanine aminotransferase (ALT), aspartate aminotransferase (AST), the AST/ALT ratio, total cholesterol, low-density lipoprotein (LDL)-cholesterol, and high-density lipoprotein (HDL)-cholesterol [7,8]. Overall, genetic studies across multiple species strongly support a role for *Agpat5* in metabolic diseases, including hyperinsulinemia, glucose intolerance, metabolic dysfunction-associated steatotic liver disease (MASLD), and dyslipidemia.

The physiological and tissue-specific roles of *Agpat5* remain understudied compared to other AGPAT enzymes. Based on the central importance of the liver in regulating glucose and lipid metabolism, we tested the liver-specific role of *Agpat5*. Our data show that liver-specific deletion of *Agpat5* does not impact plasma insulin levels, glucose tolerance, hepatic steatosis, or plasma lipid levels under multiple dietary conditions. However, when mice are supplemented with liquid sucrose—a dietary intervention that increases *de novo* lipogenesis (DNL), liver-specific *Agpat5* deletion significantly improves hyperinsulinemia and glucose intolerance. Our study demonstrates a unique pathophysiological role for *Agpat5* within the liver when consuming sucrose in liquid form without altering body weight or fat composition.

## 2. Materials and methods

### 2.1. Animal Experiments

We obtained C57BL/6 nJ-*Agpat5*^*tm1a(EUCOMM)Hmgu*^ mice from the European Conditional Mouse Mutagenesis Program, which have *loxP* sites flanking exon 6 of the *Agpat5* gene [9]. With *Agpat5 floxed* mice (*Agpat5*^*fl/fl*^), we crossed to transgenic albumin-cre (B6.Cg-Speer6-ps1^Tg(Alb-cre)21Mgn^/J – JAX #003574) mice to generate *Agpat5*^*fl/fl*^*alb-cre*^+^ and *Agpat5*^*fl/fl*^*alb-cre*^−^. Mice were interbred for littermate *Agpat5*^*fl/fl*^*alb-cre*^−^ (wild-type) and *Agpat5*^*fl/fl*^*alb-cre*^+^ (liver-specific knockout) mice. At 8 weeks of age, mice were placed on chow diet (Inotiv #2020X), high-fat lard diet (Inotiv TD.06414), Western diet (Inotiv TD.88137), or a chow diet with sucrose-sweetened water (50 % sucrose w/v). Mice were maintained on respective diets for 9 weeks and glucose tolerance tests were performed. After 12 weeks of respective diet, mice were bled after a 4-h morning fast and euthanized for tissue collection. Body composition of mice before and after respective diet intervention was performed using EchoMRI Body Composition Analyzer to quantify fat mass. Blood was collected in ethylenediaminetetraacetic acid-coated tubes (MiniCollect, 450475). All experimental procedures were performed with approval from the Institutional Animal Care and Use Committee at the University of Wisconsin-Madison.

### 2.2. Quantitative PCR

RNA was extracted in QIAzol (Qiagen #79306) and reversed transcribed with cDNA Reverse Transcription Kit (Thermo Fisher #4368813). Quantitative PCR assay was performed using KAPA-SYBRFAST (Roche #KK4611) on Roche LightCyler 480. Concentration of mRNA targets for each sample was calculated with LightCycler software based on standard curve and each target was normalized to the reference gene, *Rpl4*.

Primer sequences (forward primer, reverse primer):

*Rpl4* (AGCAGCCGGGTAGAGAGG, ATGACTCTC CCTTTTCGGAGT), *Agpat5* (ACCGGGGTCCAGATATTGCT, GCATGTCCGCAACAATCCAG)

### 2.3. Glucose tolerance tests

Mice were fasted for 6 h and administered glucose (2 g/kg) via oral gavage with glucose measured via tail blood at 0, 15, 30, 60, and 120 min with glucometer (Nova Max Plus).

### 2.4. Plasma insulin, ALT, cholesterol, and triglyceride measurements

Plasma insulin and ALT levels were measured using enzyme-linked immunosorbent assays for insulin (Alpco #80-INSMSU-EO1) and ALT (Abcam #ab282882).

### 2.5. Hepatic cholesterol and triglyceride content

Liver lipids were extracted using the Folch method [10]. Briefly, mouse liver tissue (~100 mg) was homogenized in methanol after which 2X volume of chloroform was added. Homogenates were incubated at 4 °C and the solvent was treated with 0.2X volume of 0.43 % MgCl2. Samples were allowed to settle into aqueous and organic phases, aqueous phase was removed, organic phase was dried down and resuspended in 100 % ethanol, then assayed for cholesterol (Pointe Scientific #C7510) and triglycerides (Pointe Scientific #T7532).

### 2.6. Fast protein liquid chromatography separation of mouse plasma

Size-exclusion chromatography was performed on an AKTA fastprotein liquid chromatography (FPLC) (Amersham Pharmacia). Equivalent volumes of plasma from each group of mice were pooled, totaling 500 μL. Plasma was applied to a Superose 6 followed in tandem with a Superdex 200 column and separated in 10 mM PBS, pH 7.4, and collected into 48, 0.5 ml fractions. Fractions were assayed for cholesterol content (Pointe Scientific #C7510).

### 2.7. Liver lipid extraction for mass spectrometry

21 (± 2) mg of liver was mixed with 250 μL master mix of ice-cold water and methanol containing mixture of internal standards (10 μL of SPLASH, 300 pmol oleoyl-carnitine-d3 and 300 pmol ceramide (d18:1-d7/15:0) per sample) and homogenized in ceramic 1.4 mm bead tubes (Omni International, #19–627) using the Qiagen TissueLyzer II (#9244420) for two cycles using chilled (4°C) blocks. The resulting homogenates were mixed with ice-cold water and 750 μL ice-cold methyl *tert*-butyl ether and shaken in Qiagen TissueLyzer II for one cycle. Samples were centrifuged at 16,000 g (4°C for 10min) to induce phase separation. The upper phase was collected and dried using a SpeedVac with nitrogen flow. Lipids were resolubilized in 100 % isopropanol. For positive ionization mode, isopropanol extracts were diluted 120x with 100 % isopropanol. For negative ionization mode undiluted isopropanol extracts were analyzed.

### 2.8. Liquid chromatography tandem mass spectrometry analysis

Samples were injected on an Agilent 1260 Infinity II Ultra High-Performance Liquid Chromatography system connected to Agilent 6546 quadrupole time-of-flight mass spectrometer (MS) dual Agilent Jet Stream electrospray ionization MS and analyzed in positive and negative mode. Injected volumes were 3 μL in the positive mode and 5 μL in the negative mode. Lipids were separated by an Acquity Bridged Ethyl-Siloxane/Silica Hybrid (BEH) C_18_ column (Waters #186002352; 1.7 μM 2.1 × 100 mm) at 50 °C with VanGuard BEH C_18_ precolumn (Waters #18003975). A binary solvent system of mobile phase A (acetonitrile: water (60:40, v/v)) and mobile phase B (isopropanol:acetonitrile:water (90:9:1, v/v/v)), both containing 10 mM ammonium formate, and 0.1 % formic acid. Separation achieved with a flow rate of 0.5 mL/min, for a total of 20 min, with the following gradient: 0 min, 15 % B; 0–2.4 min, 15–30 % B; 2.4–3 min, 30–48 % B; 3–13.2 min, 48–82 % B; 13.2–13.8, 82–99 % B; 13.8–15.4, 99 % B; followed by 4.2 re-equilibration to 15 %. MS conditions used in positive mode were: gas temperature: 250 °C; drying gas rate: 12 L/min; nebulizer: 35 psi; sheath gas temperature: 300 °C; sheath gas rate: 11 L/min. For negative mode the gas temperature and drying gas rate were the same as in the positive mode, whereas the nebulizer was kept at 30 psi, sheath gas temperature at 375 °C and sheath gas flow at 12 L/min. The Vcap voltage was set at 4000 V, skimmer at 75 V, fragmentor at 190 V, and octupole radiofrequency peak at 750 V for both ionization modes. Samples were analyzed as single batch, injected in randomized order, and scanned from mass-to-charge ration (*m/z*) 100 to 1700. Mass correction was implemented during data acquisition using 2 and 3 reference masses in positive and negative ionization mode, respectively (*m/z* 121.05 and 922.00 in positive mode; *m/z* 112.98, 966.00 and 1033.99 in negative mode). Tandem MS was performed at fixed collision energy of 25 V.

### 2.9. Mass spectrometry data processing

Raw data uploaded to MassIVE repository (#MSV000096473). Quantification performed with Agilent MassHunter Workstation v11 and normalized to the relative concentration of internal standard spiked into each sample pre-extraction. Volcano plots and bar graphs were made using the ggpubr package in R (R version 4.1.2). Data were normalized to protein content with bicinchoninic acid assay (Thermo Scientific 23225) in the aqueous phase following lipid extraction. Data reported in ng lipid/μg protein.

### 2.10. Statistical analysis

Statistical analysis of genotype effects was performed using student’s unpaired two-tailed t-tests between two groups. Statistical significance was defined as follows. *p < 0.05, **p < 0.01, and ***p < 0.001. Data presented as mean ± standard deviation with individual values. Analysis performed using GraphPad prism.

## 3. Results

### 3.1. Genetic associations with Agpat5

In mice and humans, there is strong genetic data supporting a role for *Agpat5* in contributing to hyperinsulinemia, glucose metabolism, MASLD, and plasma lipid metabolism. The first study to report an association for *Agpat5* used the hybrid mouse diversity panel, a mouse genetic reference population (GRP), that was fed a high-fat/high-sucrose diet. The study identified a genome-wide significant locus on mouse chromosome 8 associated with plasma insulin levels, which contained *Agpat5* [5]. The second study used the BXD (C57BL/6J (B6) mice and DBA/2J (D2) mice) recombinant inbred GRP and identified the same locus on chromosome 8 associated with hypoglycemia-induced glucagon secretion [6]. These studies indicate that *Agpat5* may contribute to hyperinsulinemia and glucose metabolism.

**Fig. 1.**
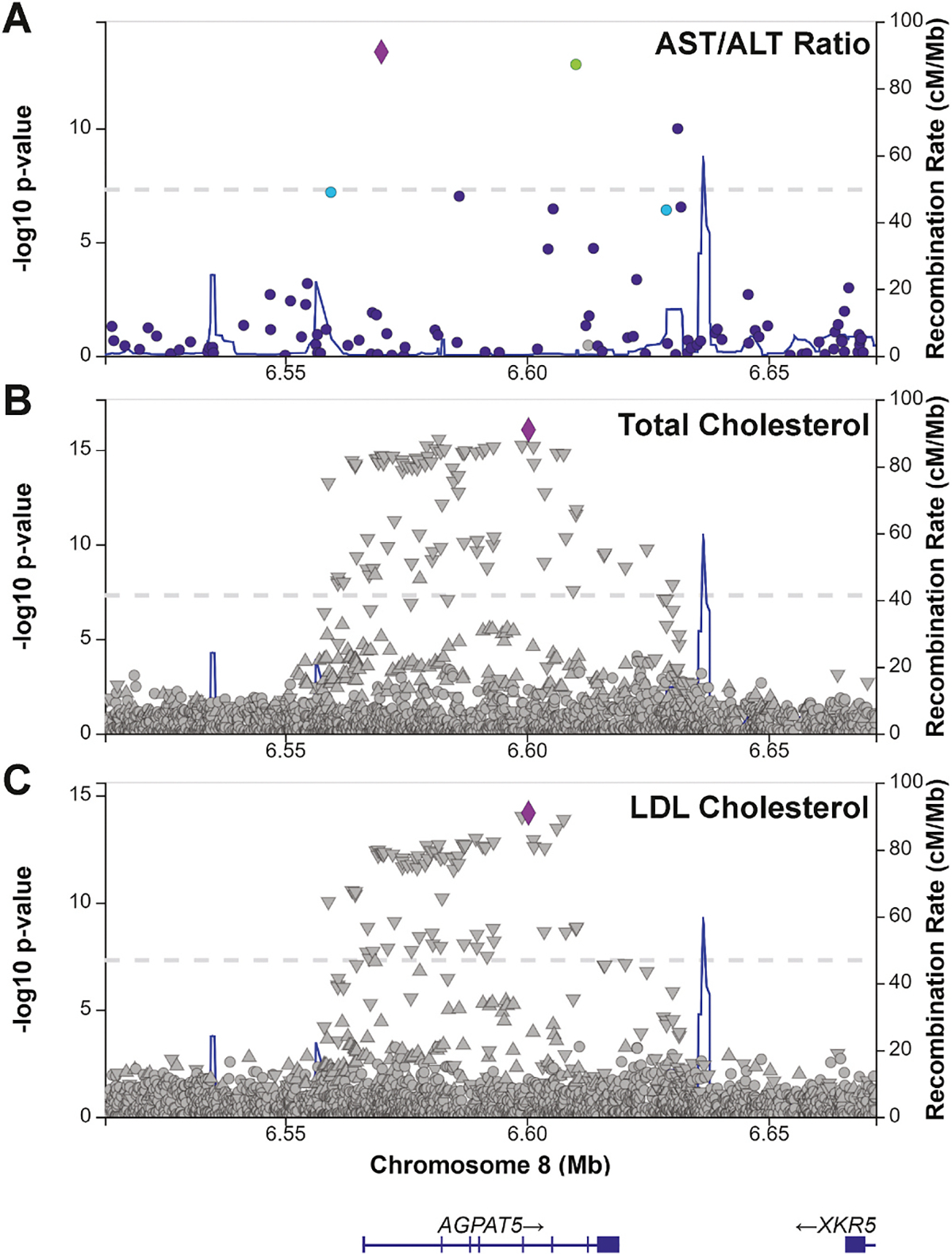
AGPAT5 is associated with plasma AST/ALT ratio and plasma lipids in humans. Regional association plots of AGPAT5 with (A) AST/ALT ratio in the Million Veteran Program 2024 dataset (B) total cholesterol, and (C) low-density lipoprotein (LDL)-cholesterol in the Global Lipid Genetics Consortium (GLGC) 2021 dataset. The left y-axis shows significance of association and the right y-axis shows the recombination rate across the region (blue line). The purple diamond indicates the most associated single nucleotide polymorphism (SNP).

Multiple large-scale human GWAS have identified *AGPAT5*. In the Million Veterans Program genetics study, *AGPAT5* is contained within a genome-wide significant locus associated with plasma ALT, AST, and AST/ALT ratio (Fig. 1A) [8]. Elevated levels of ALT and AST serve as indicators of MASLD, suggesting that *AGPAT5* may contribute to this condition [11]. Additionally, a GWAS of plasma lipids in 1.65 million individuals identified *AGPAT5* contained within a genome-wide significant locus associated with plasma levels of total cholesterol, LDL-cholesterol, and HDL-cholesterol (Fig. 1B and C) [7]. This genetic data coupled with the evidence that *AGPAT5* expression within liver tissue of humans and mice is under significant local regulation (*cis* eQTL), suggests *AGPAT5* may operate at the level of the liver [5,12]. Given the significant genetic findings and the unknown role of *AGPAT5* in the liver, we investigated liver-specific function in mice.

### 3.2. Liver Agpat5 contributes to hyperinsulinemia and glucose intolerance

**Fig. 2.**
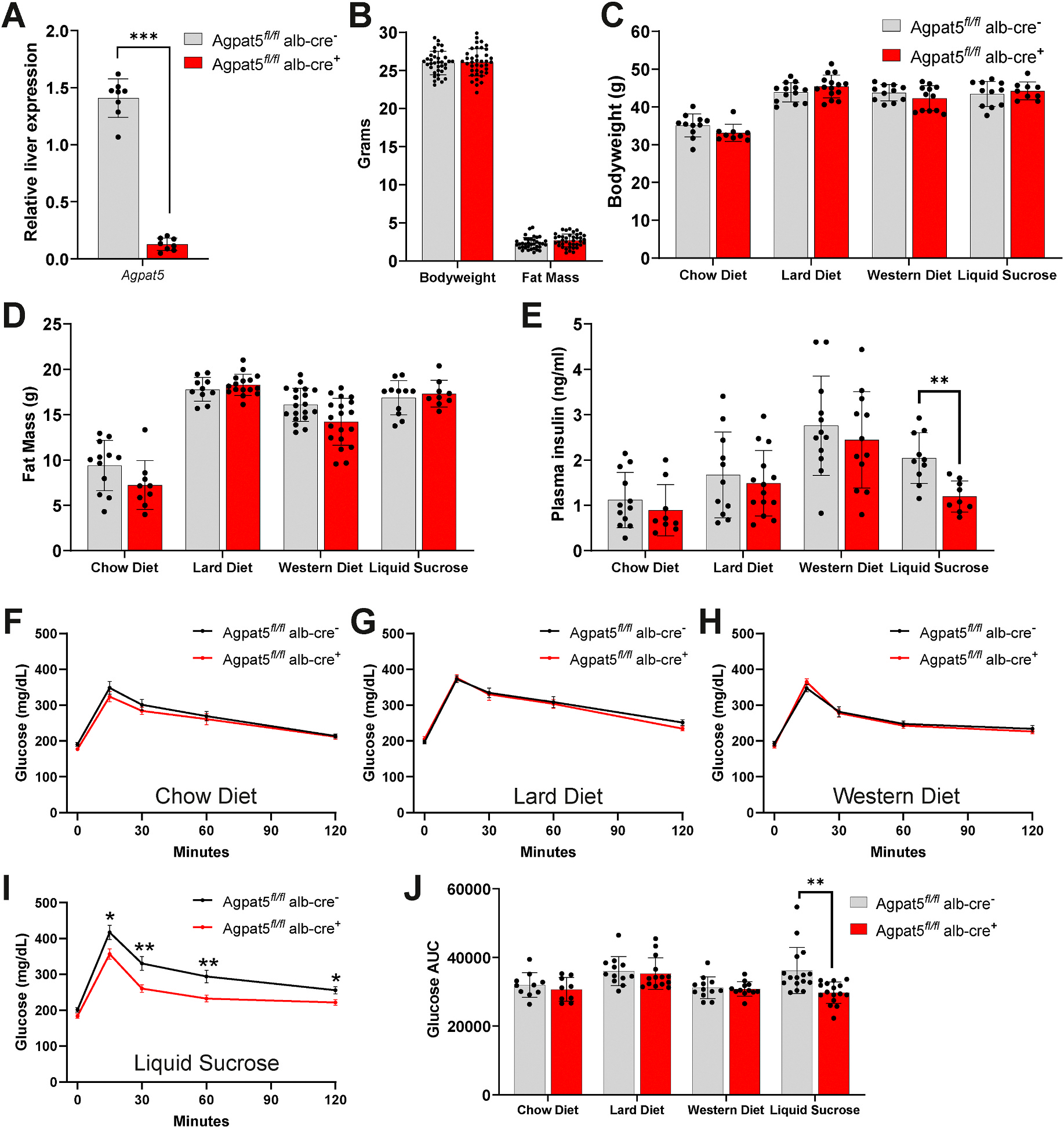
Liver-specific Agpat5 deletion improves hyperinsulinemia and glucose intolerance. Agpat5 fl/fl.alb-cre − (Wild-type) and Agpat5 fl/fl alb-cre + (Liver-specific knockout) mice (A) relative gene expression of Agpat5 in the liver (B) bodyweight and fat mass at 8 weeks of age. (C) Bodyweight, (D) fat mass, and (E) plasma insulin levels in Agpat5 fl/fl alb-cre − and Agpat5 fl/fl alb-cre + mice after 12 weeks of feeding a chow diet, lard diet, western diet, or chow diet with liquid sucrose. Glucose tolerance test (GTT) in Agpat5 fl/fl alb-cre − and Agpat5 fl/fl alb-cre + mice fed a (F) chow diet (G) lard diet (H) Western diet, or (I) chow diet with liquid sucrose for nine weeks. (J) Area under curve (AUC) measure from GTT from time 0–120 min in Agpat5fl/fl.alb-cre − and Agpat5 fl/fl alb-cre + mice fed a chow diet, lard diet, western diet, or chow diet with liquid sucrose. Data presented as mean ± standard deviation with individual mice represented. N > 8 for each genotype and condition. *P < 0.05, **P < 0.01, ***P < 0.001.

To investigate the liver-specific role of *Agpat5*, we developed a *floxed* mouse for *Agpat5* (*Agpat5*^*fl/fl*^), with *loxp* sites flanking exon 6, which regulates membrane binding. *Agpat5*^*fl/fl*^ mice were crossed with Albumin *cre*-recombinase mice (*alb-cre*) to delete *Agpat5* within the liver and were interbred to generate littermate wild-type control mice (*Agpat5*^*fl/fl*^*alb-cre*^−^) and liver-specific knockout mice (*Agpat5*^*fl/fl*^*alb-cre*^+^). Expression of *Agpat5* in the liver was significantly reduced in *Agpat5*^*fl/fl*^*alb-cre* + mice compared to *Agpat5*^*fl/fl*^*alb-cre*^−^ mice (Fig. 2A). At 8 weeks of age, mice were placed onto four distinct dietary interventions, control chow diet, high-fat lard diet (60%fat), Western diet (42 % fat), and a chow diet supplemented with liquid sucrose drinking water (50 % w/v sucrose) for 12 weeks. These diets were selected to disrupt lipid homeostasis by increasing fatty acid levels, increasing DNL, and contributing to metabolic disease. Specifically, the liquid sucrose supplementation strongly promotes DNL while also increasing calorie intake, body weight gain, and hepatic steatosis. Before starting their diet at 8 weeks and after 12 weeks of intervention, there were no differences in body weight or fat mass between *Agpat5*^*fl/fl*^*alb-cre*^−^ and *Agpat5*^*fl/fl*^*alb-cre* + mice (Fig. 2B–D). After 12 weeks of dietary intervention, there was no difference in plasma insulin levels in the chow diet, lard diet, or Western diet groups, but *Agpat5*^*fl/fl*^*alb-cre* + mice fed a chow diet with liquid sucrose had significantly reduced plasma insulin compared to *Agpat5*^*fl/fl*^*alb-cre*^−^ mice (Fig. 2E).

We also tested the impact of liver-specific *Agpat5* on glucose tolerance after 9 weeks of diet for each of the four dietary interventions. Both *Agpat5*^*fl/fl*^*alb-cre*^−^ and *Agpat5*^*fl/fl*^*alb-cre* + mice had similar glucose tolerance levels throughout the time course of the glucose tolerance test (GTT) when fed a chow diet, lard diet, or Western diet (Fig. 2F–H). In mice fed a chow diet with liquid sucrose, *Agpat5*^*fl/fl*^*alb-cre* + mice showed significantly reduced levels of glucose at 15, 30, 60,and 120 min during the GTT (Fig. 2I). Consistent with reduced levels of glucose during the GTT, *Agpat5*^*fl/fl*^*alb-cre* + mice fed a chow diet with liquid sucrose were the only dietary intervention found to have a significantly reduced area under the curve (Fig. 2J). Overall, these data show that liver-specific deletion of *Agpat5* significantly improves the development of hyperinsulinemia and glucose intolerance in mice on chow diet with liquid sucrose without changing body weight or fat mass.

### 3.3. Liver Agpat5 does not impact hepatic steatosis or plasma lipid levels

**Fig. 3.**
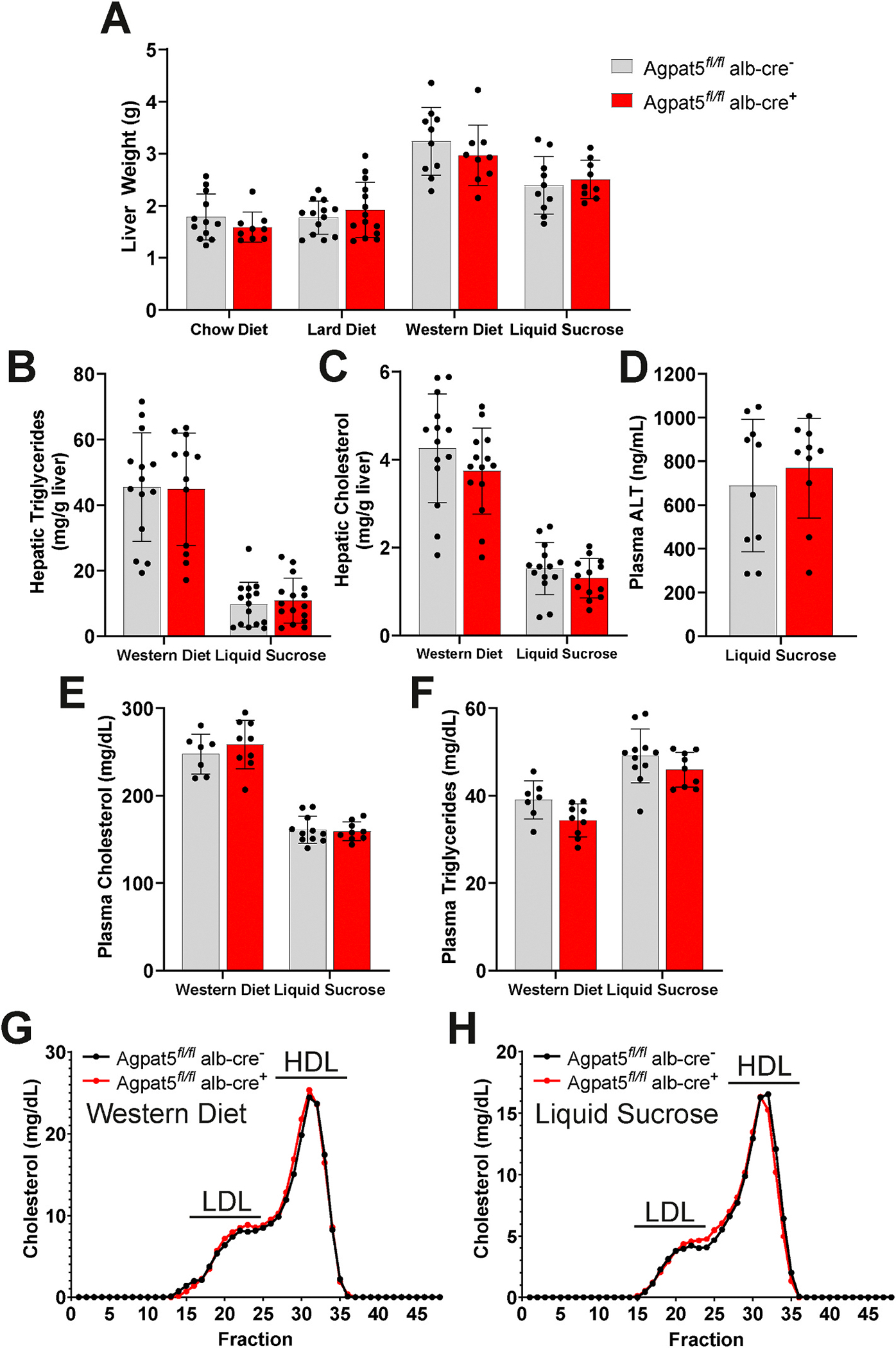
Liver-specific Agpat5 does not impact hepatic steatosis or plasma lipid levels. (A) Total liver weight of Agpat5 fl/fl alb-cre − (Wild-type) and Agpat5 fl/fl alb-cre + (Liver-specific knockout) mice after 12 weeks of feeding a chow diet, lard diet, western diet, or chow diet with liquid sucrose. Liver (B) triglyceride and (C) cholesterol content in Agpat5 fl/fl alb-cre − and Agpat5 fl/fl alb-cre + mice fed a Western diet and chow diet with liquid sucrose for 12 weeks. (D) Plasma alanine aminotransferase (ALT) levels in Agpat5 fl/fl alb-cre − and Agpat5 fl/fl alb-cre + mice fed a chow diet with liquid sucrose for 12 weeks. Total plasma levels of (E) cholesterol and (F) triglycerides in Agpat5 fl/fl alb-cre − and Agpat5 fl/fl alb-cre + mice fed a Western diet and chow diet with liquid sucrose for 12 weeks. Plasma fractionation in mice fed a (G) Western diet and (H) chow diet with liquid sucrose for 12 weeks. LDL: slow-density lipoprotein (LDL)-cholesterol; HDL: high-density lipoprotein (HDL)-cholesterol. Data presented as mean ± standard deviation with individual mice represented. N > 10 for each genotype and condition.

To investigate the effect of liver-specific Agpat5 deletion, we also measured hepatic steatosis and plasma lipid levels among the four dietary interventions. Liver weight did not differ between *Agpat5*^*fl/fl*^*alb-cre*^−^ and *Agpat5*^*fl/fl*^*alb-cre* + mice on chow diet, lard diet, Western diet, or chow diet with liquid sucrose-fed mice (Fig. 3A). Since the Western diet and chow diet supplemented with liquid sucrose groups resulted in the largest liver weights, we selected those groups for further analysis. Hepatic steatosis was assessed by quantifying triglycerides and cholesterol in the liver, but no significant differences were observed between *Agpat5*^*fl/fl*^*alb-cre*^−^ and *Agpat5*^*fl/fl*^*alb-cre* + mice (Fig. 3B and C). Furthermore, in chow diet mice with liquid sucrose, which showed improvements in insulin levels and glucose intolerance *Agpat5*^*fl/fl*^*alb-cre*^−^ and *Agpat5*^*fl/fl*^*alb-cre* + mice had no differences in plasma ALT levels (Fig. 3D). Total plasma cholesterol and triglyceride levels were also similar in both groups of mice fed a Western diet and chow diet supplemented with liquid sucrose (Fig. 3E and F). To distinguish specific effects of *Agpat5* on LDL-cholesterol versus HDL-cholesterol, we performed FPLC on plasma in *Agpat5*^*fl/fl*^*alb-cre*^−^ and *Agpat5*^*fl/fl*^*alb-cre* + mice fed a Western diet and chow diet with liquid sucrose and detected no differences (Fig. 3G and H). These data indicate that liver-specific deletion of *Agpat5* does not contribute to hepatic steatosis or influence plasma levels of LDL-cholesterol or HDL-cholesterol.

### 3.4. Specific glycerophospholipids regulated by Agpat5 in the liver

**Fig. 4.**
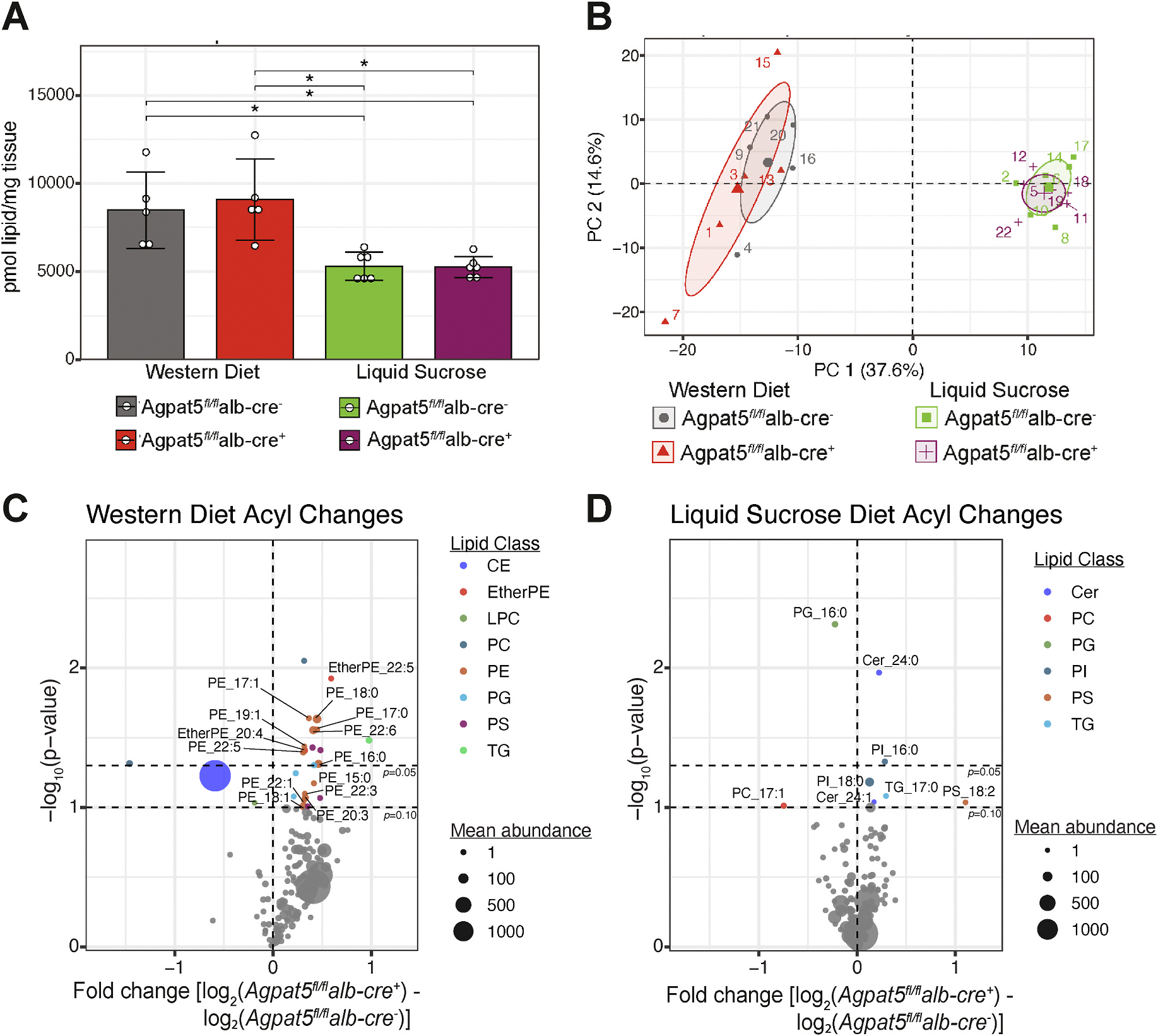
Specific glycerophospholipids regulated by Agpat5 in the liver. Global lipidomics analysis of Agpat5 fl/fl alb-cre − (Wild-type) and Agpat5 fl/fl alb-cre + (Liver-specific knockout) mice fed a Western diet or chow diet with liquid sucrose for 12 weeks. (A) Total liver lipids quantified (B) Principal component analysis (PCA) of first two principal components (PC1; PC2) (C) Volcano plot of altered lipid acyl species in (C) Western diet-fed mice and (D) chow diet supplemented with liquid sucrose mice CE: cholesterol ester species; EtherPE: ether phatidylethanolamine species; LPC: lysophosphatidylcholine species; PC: phosphatidylcholine species; PE: phosphatidylethanolamine species; PG: phosphatidylglycerol species; PS: phosphatidylserine species; TG: triglyceride species; PI: phosphatidylinositol species. N = 5 mice for each genotype and condition. *P < 0.05 unless otherwise stated.

Within the AGPAT family of enzymes, *Agpat5* has broad substrate specificity, acylating the *sn*-2 position of diverse glycerophospholipid species (*e.g.,* LPA, LPE, LPC, LPI, LPS) [4]. To investigate how *Agpat5* impacts specific lipid class changes in the liver, we performed global lipidomics in *Agpat5*^*fl/fl*^*alb-cre*^−^ and *Agpat5*^*fl/fl*^*alb-cre* + mice fed either a Western diet or a chow diet supplemented with liquid sucrose. Total lipid levels were similar between the two groups under both dietary conditions, although liquid sucrose-fed mice showed significantly lower total lipid levels (Fig. 4A). These total lipid levels are consistent with quantification of triglycerides and cholesterol (Fig. 3B and C), which showed lower lipid levels in chow-fed supplemented with liquid sucrose in comparison to Western diet-fed mice. Principal component analysis revealed a clear separation of lipid classes between mice on a Western diet and those on the chow diet supplemented with liquid sucrose (Fig. 4B). While liver-specific deletion of *Agpat5* did not alter the overall abundance of lipid classes, it significantly affected specific lipid species. In Western diet-fed mice, 23 lipid species were significantly increased, and 3 were significantly decreased in *Agpat5*^*fl/fl*^*alb-cre* + compared to *Agpat5*^*fl/fl*^*alb-cre*^−^ mice (Supplemental Table 1). Focusing on acyl chain abundance revealed that the lipids increased in *Agpat5*^*fl/fl*^*alb-cre* + mice fed a Western diet were primarily long-chain and very long-chain PE species (Fig. 4C). In chow diet mice supplemented with liquid sucrose, 5 lipid species were significantly increased, and 3 were significantly decreased in *Agpat5*^*fl/fl*^*alb-cre* + mice (Supplemental Table 2). These lipids include signaling lipids, such as ceramides (CE) and phosphatidylinositol (PI), as well as lipids found in high abundance in the mitochondria, including cardiolipin and phosphatidylglycerol (PG)—an interesting observation given that others have observed localization of *Agpat5* to the mitochondria [4]. Acyl chain analysis revealed that all significantly altered lipids in the *Agpat5*^*fl/fl*^*alb-cre* + mice fed a chow diet supplemented with liquid sucrose contained saturated acyl chains (Fig. 4D). Notably, levels of PG 16:0/18:1 were significantly reduced across both dietary conditions, suggesting that *Agpat5* may regulate PG biosynthesis through its acyltransferase activity. Our global lipidomic analysis highlights that *Agpat5* plays a specific role in glycerophospholipid metabolism in the liver, selectively influencing certain lipid species without broadly affecting overall lipid class abundance.

## 4. Discussion

Genetic evidence strongly supports a role for *Agpat5* in contributing to hyperinsulinemia, glucose intolerance, hepatic steatosis, and dyslipidemia. Previous studies also highlight a role for *Agpat5* in regulating insulin and glucose metabolism. One study showed that targeting *Agpat5* in mice with antisense oligonucleotides improves hyperinsulinemia and glucose intolerance [5]. Another study demonstrated that expression of *Agpat5* in agouti-related peptide neurons in the hypothalamus contributes to hypoglycemia-induced glucagon secretion [13]. However, our study is the first to demonstrate the contribution of *Agpat5* in the liver. We show that liver-specific expression of *Agpat5* contributes to hyperinsulinemia and glucose intolerance only in mice consuming a chow diet supplemented with liquid sucrose drinking water. Liver-specific deletion of *Agpat5* had no effect on body weight, fat mass, hepatic steatosis, or plasma levels of LDL-cholesterol and HDL-cholesterol in any of the four dietary interventions.

Liver-specific deletion of *Agpat5* improved plasma insulin levels and glucose intolerance only in mice fed a chow diet supplemented with liquid sucrose. Ingestion of sucrose in the liquid form versus solid form strongly contributes to obesity, glucose intolerance, and hepatic steatosis in mice—specifically driving an increase in DNL [14,15]. As sucrose is a disaccharide composed of glucose and fructose, mice consuming liquid sucrose would intake large amounts of fructose. In the liver, fructose metabolism is not regulated and depletes adenosine triphosphate (ATP) levels [16]. This coupled with data showing silencing *Agpat5* increases cellular ATP levels suggests that liver-specific *Agpat5* knockout mice may be able to better regulate ATP levels when consuming excess fructose [13]. This concept is consistent with the observation that impaired ATP homeostasis within the liver is a contributing factor to insulin sensitivity in patients with type 2 diabetes [17].

Liver-specific deletion of *Agpat5* did not affect body weight or fat mass and yet still had improved plasma insulin levels and glucose intolerance when fed a chow diet supplemented with liquid sucrose. This separation of body weight and fat mass from insulin and glucose intolerance is unique. Through global lipidomic profiling, we show liver-specific *Agpat5* knockout mice fed a chow diet supplemented with liquid sucrose only influenced 8 lipid species. One of the significantly increased lipid species is the glycerophospholipid, cardiolipin (CL) 74:10. CL plays an important role in contributing to mitochondrial function and alterations in CL can influence liver insulin sensitivity [18]. This change in CL 74:10 along with the reduced levels of PG 16:0/18:1 may indicate that *Agpat5* alters lipid metabolism in the mitochondria. Furthermore, this data suggests that *Agpat5* may contribute to tissue insulin sensitivity through specific changes in lipid species within the liver.

Our study provides strong evidence that in the liver *Agpat5* contributes to hyperinsulinemia and glucose intolerance when consuming liquid sucrose by altering specific glycerophospholipids. Further work is needed to establish if liver loss of *Agpat5* is buffered through compensation by other AGPAT enzymes, including *Agpat4*, which is also mitochondrially localized. Finally, establishing models with tissue-specific loss of *Agpat5* opens the door for targeted exploration of human polymorphisms of *AGPAT5* which may contribute to risk of MASLD.

## Supplementary Material

MMC2

MMC1
